# Evidence for Cohesive Dispersal in the Sea

**DOI:** 10.1371/journal.pone.0042672

**Published:** 2012-09-18

**Authors:** Ofer Ben-Tzvi, Avigdor Abelson, Steven D. Gaines, Giacomo Bernardi, Ricardo Beldade, Michael S. Sheehy, Georges L. Paradis, Moshe Kiflawi

**Affiliations:** 1 The Interuniversity Institute for Marine Sciences of Eilat, Eilat, Israel; 2 Department of Zoology, George S. Wise Faculty of Life Sciences, Tel-Aviv University, Tel-Aviv, Israel; 3 Bren School of Environmental Science and Management, University of California Santa Barbara, Santa Barbara, California, United States of America; 4 Department of Ecology and Evolutionary Biology; University of California Santa Cruz, Santa Cruz, California, United States of America; 5 Department of Earth Science, University of California Santa Barbara, Santa Barbara, California, United States of America; 6 Department of Life Sciences, Ben-Gurion University, Be'er Sheva, Israel; University of Hamburg, Germany

## Abstract

As with many marine species, the vast majority of coral-reef fishes have a bipartite life cycle consisting of a dispersive larval stage and a benthic adult stage. While the potentially far-reaching demographic and ecological consequences of marine dispersal are widely appreciated, little is known of the structure of the larval pool and of the dispersive process itself. Utilizing Palindrome Sequence Analysis of otolith micro-chemistry (PaSA;) we show that larvae of *Neopomacentrus miryae* (Pomacentridae) appear to remain in cohesive cohorts throughout their entire pelagic larval duration (PLD; ∼28 days). Genetically, we found cohort members to be maternally (mtDNA) unrelated. While physical forcing cannot be negated as contributing to initial cohort formation, the small scale of the observed spatial structure suggests that some behavioral modification may be involved from a very early age. This study contributes to our ongoing re-evaluation of the processes that structure marine populations and communities and the spatial scales at which they operate.

## Introduction

Upon hatching, the larvae of most coastal marine species (including those having benthic eggs) may be passively advected considerable distances before settling back onto the benthos [1]. Such dispersal is commonly modelled as a diffusive process that mixes larvae from different source locations; gradually erasing, within the larval pool, any spatial patterns introduced by adult-population structure [2], [3]. Realized patterns of dispersal, however, may be quite different, with far reaching demographic, ecological and micro-evolutionary consequences [4], [5], [6].

Several mechanisms can prevent larvae from slowly being mixed into a diffuse pool. For example, the number of independent dispersal trajectories may be limited by spatial autocorrelation in the movement of water masses [6], [7] and/or spatial heterogeneity in the conditions necessary for larval survival [8]. Alternatively, spatial variation in spawning success could render the larval pool itself a non-random sample of the adult population (“reproductive sweepstakes”) [9]. Finally, larval behaviour is increasingly invoked as another mechanism that could significantly modify passive, diffusive transport [10]. Evaluation of the mechanisms that could potentially structure the larval pool is hampered, however, by a dearth of direct ‘observations’ of the paths followed by marine larvae as they disperse [11].

For coral-reef and other coastal demersal fishes, evidence for constrained dispersal and demographic connectivity are commonly sought in patterns of population genetic structure [12]. However, the spatial and temporal resolution of these patterns is commonly too coarse to expose the underlying mechanisms. Consequently, biologists and fishery scientists are making increasing use of the micro-chemical information stored in the otoliths of larval fish [13], [14], [15]. Otoliths, calcareous structures that form part of the auditory-vestibular system, are characterized by a clearly visible pattern of daily growth. To date, otolith micro-chemistry has been used to reconstruct ontogenetic habitat shifts (e.g. estuary; rivers; open sea), evaluate local retention and connectivity, and identify stocks and source-populations [16], [17]. However, successful application of this approach has only been achieved at relatively coarse spatial scales.

Attempts to track the dispersal path of individual larvae make use of trace-element sequences generated using Laser Ablation Inductively Coupled Plasma Mass Spectrometry (LA-ICPMS) [18], [19]. Sequences that span the section of the otolith that corresponds to the larval stage provide a chronological record of the microenvironment experienced by individual larvae during dispersal [20]. The next step of matching a signal in the otolith to specific geographic locations, however, has proven elusive for a variety of complications [21].

Here we take a different approach to glean insight from otoliths' microchemistry sequences. Our approach does not require inferences regarding the geographic location of the larvae at any particular point during its PLD. Rather, we view statistically-significant sequence similarity among cohort members (see below for definition) as a reasonable indication of shared dispersal paths; even when little is known of the path itself. Inferring shared paths from shared otolith chemistry (i.e. from intra-cohort similarities that exceed the expectation by chance) assumes that the environment through which the cohorts have been moving is chemically heterogeneous, at the relevant scale. The longer the shared microchemical sequence, the longer the larvae are likely to have been together. Moreover, by combining the micro-chemical and chronological information found in their otoliths, the point along the pelagic duration at which cohort members began to share a common path can be deduced. With a purely diffusive larval pool we would expect cohort members to follow highly independent paths and, even when moving through a chemically heterogeneous environment, show a level of intra-cohort similarity that is indistinguishable from chance alone.

We applied this approach in the analyses of several cohorts of two coral-reef fish: *Neopomacentrus miryae* and *Chromis viridis* (Cuvier, 1830). The first species is endemic to the Red-Sea and Gulf of Aden and forms large schools near vertical structures in the reef (e.g. reef walls and knolls). Larvae hatch from demersal eggs (documented for the congener *N. taeniurus*; [22] and observed once for *N. miryae* by O.B.Z.) and settle mainly between November and April in large schools (Ben-Tzvi, personal observations). The second species is a coral-dwelling damselfish of Indo-Pacific distribution that, in the Gulf of Aqaba, reproduces between June and February, lays demersal eggs and settles after 22–26 days [18]. In the context of this study, we define a “cohort” as a group of same-aged fish that settled, on the same night, onto the same highly localized site (e.g. small knoll; coral colony).

## Results

Based on otoliths' data, the 40 *N. miryae* that were sampled belonged to one of 6 cohorts (Table 1). Of the 34 *C. viridis* sampled, only 16 could be assigned to one of five cohorts (Table 2); with the remaining fish being of different hatching dates.

**Table 1:** Similarity matrix of trace-element sequences for six cohorts of *N. miryae*.

	IUI a (28, 14)	IUI b (28, 4)	IUI c (29, 3)	PB a (29, 5)	PB b (29, 7)	NB (29, 7)	Expected
IUI a (08\12\03)	**0.80 (0.16)**						0.41–0.50
IUI b (16\03\04)	0.37 (0.06)	**0.94 (0.06)**					0.30–0.66
IUI c (15\03\04)	0.37 (0.05)	0.36 (0.13)	**0.89 (0.09)**				0.29–0.66
PB a (16\03\04)	0.35 (0.09)	0.44 (0.06)	0.37 (0.10)	**1.00 (0.00)**			0.36–0.60
PB b (22\03\04)	0.40 (0.06)	0.34 (0.06)	0.25 (0.09)	0.35 (0.06)	**0.97 (0.05)**		0.39–0.55
NB (26\03\04)	0.37 (0.07)	0.29 (0.08)	0.49 (0.11)	0.33 (0.07)	0.28 (0.06)	**0.90 (0.09)**	0.38–0.57

Each cell contains the mean similarity (± standard deviation) of sequences that roughly correspond to the first half the PLD. Row headings provide each cohort's name and back-calculated hatch date. Column headings provide, in parentheses, the cohort's PLD and sample size. The 5^th^ & 95^th^ percentiles of the expected (by chance) mean for cohort similarity are presented in the far right column (see Methods for details). Highlighted are similarity values that fall above the expected 95^th^ percentile (right figure of the far right column). The IUI is situated 6 km south of NB, with PB 1 km further south (lower case letters distinguish between multiple cohorts collected in any one of these sites).

**Table 2:** Similarity matrix of trace-element sequences for five cohorts of *C. Viridis*.

	C1 (26, 5)	C2 (26, 3)	C3 (26, 3)	C4 (26, 2)	C5 (26, 3)	Expected
C 1 (15\10\05)	0.28 (0.1)					0.29–0.35
C 2 (17\10\05)	0.30 (0.08)	0.29 (0.04)				0.26–0.38
C 3 (13\10\05)	0.32 (0.08)	0.33 (0.09)	0.39 (0.06)			0.28–0.39
C 4 (16\10\05)	0.29 (0.05)	0.37 (0.10)	0.34 (0.05)	0.28 (0.00)		0.23–0.44
C 5 (14\10\05)	0.33 (0.11)	0.38 (0.08)	0.32 (0.05)	0.32 (0.08)	0.34 (0.06)	0.28–0.39

Each cell contains the mean similarity (± standard deviation) of sequences that roughly correspond to the first two-thirds of the PLD. Row headings provide each cohort's name and back-calculated hatch date. Column headings provide, in parentheses, the cohort's PLD and sample size. The 5^th^ & 95^th^ percentiles of the expected (by chance) mean for cohort similarity are presented in the far right column (see Methods for details). Highlighted are similarity values that fall above the expected 95^th^ percentile (right figure of the far right column). All cohorts of *C. viridis* were collected from a single coral colony, at the IUI.

**Figure 1:**
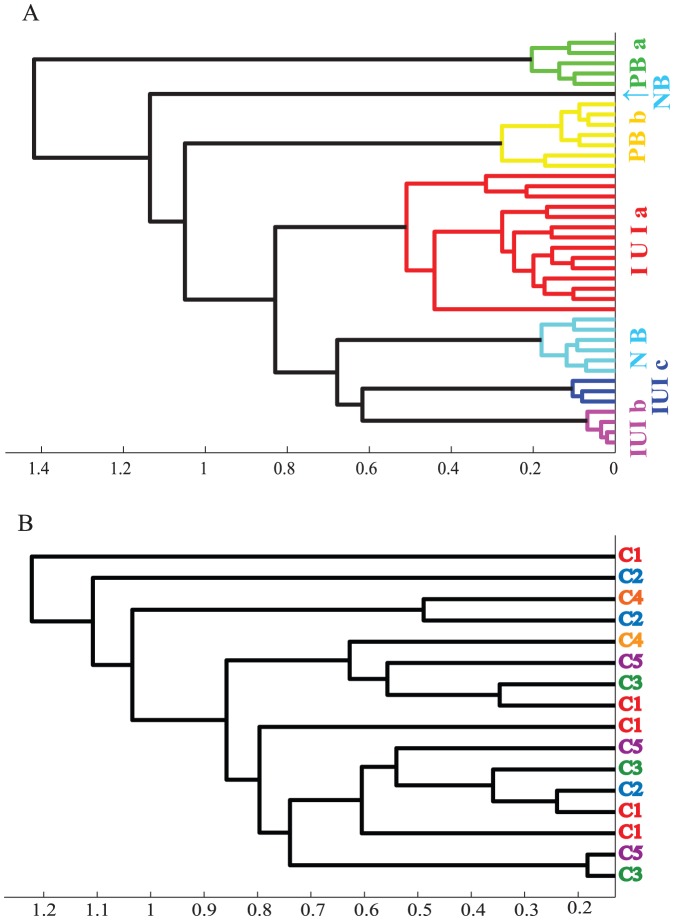
Hierarchical cluster analysis of cohort members, based on standardized trace-element concentrations in their otoliths' core. Clustering of 40 *N. miryae* recruits collected at 6 different times and/or locations, and 16 *C. viridis* recruits collected from the same coral head. Cohort abbreviations are as in Table 1 and 2. Classification is based on Euclidean distances and average linkage, and provides a faithful representation of the structure in the original data (Cophenetic correlations: 0.94 and 0.78 for *N. Miryae* and *C. viridis*; respectively).

In terms of otolith microchemistry, the findings for the two species were strikingly different. For *N. miryae*, members of a given cohort had nearly identical trace-element sequences with average sequence similarities ranging between 0.8 and 1.0 (Table 1). These high similarities far exceed what would be expected by chance alone (Table 1). They also greatly exceed the similarities observed between members of different cohorts (range: 0.25 to 0.49). Inter-cohort similarity levels were low even when cohorts settle within days or short distances of each other. Especially informative were two cohorts that settled at the same site after temporally overlapping for all but one day of their PLD (IUIb&c). Focusing on the elemental signature of the otoliths' core we also found that cohort members strongly cluster (Fig. 1A); indicating that cohort members originated from the same locality [14], [15] and proceeded to share a similar dispersal path. The one inconsistency involves a single NB recruit that had Mg concentrations, throughout the otolith, an order of magnitude higher than all of the rest. This individual was included in the analysis of sequence similarity which, due to lack of significant within-otolith variation, does not consider Mg.

For *C. viridis*, the patterns in otolith microchemistry were strikingly different; despite of showing similar levels of variation in trace-element concentrations as *N. miryae* (Table 3). Specifically, both within and among cohort similarities, in trace-element sequences were consistently low (all less than 0.38), and never differed from the null expectation (Table 2). Moreover, cohort members did not cluster according to core microchemistry in any meaningful way (Fig. 1B).

**Table 3:** Otolith concentrations of six trace-elements.

	Mg/Ca mmol/mol	Cr/Ca µmol/mol	Mn/Ca µmol/mol	Cu/Ca µmol/mol	Sr/Ca µmol/mol	Pb/Ca µmol/mol
*Cv* core	0.20±0.09	0.64±1.20	144.47±87.18	1.03±1.42	2.92±0.39	0.15±0.36
*Cv* seq		2.95±1.72	3.4±1.21	3.55±1.83		0.79±0.61
*Nm* core	0.33±0.10	0.86±0.76	316.87±174.33	1.31±1.72	2.69±0.22	0.15±0.33
*Nm* seq		3.30±1.89	10.37±6.92	4.36±2.26		0.87±0.57

Mean (± SD) relative concentrations in the cores and along the pre-settlement regions (seq.) of the otoliths of *C. viridis* (*Cv*) and *N. miryae* (*Nm*).

The mean and SD for the pre-settlement region relate only to those readings that make up the palindrome.

**Figure 2:**
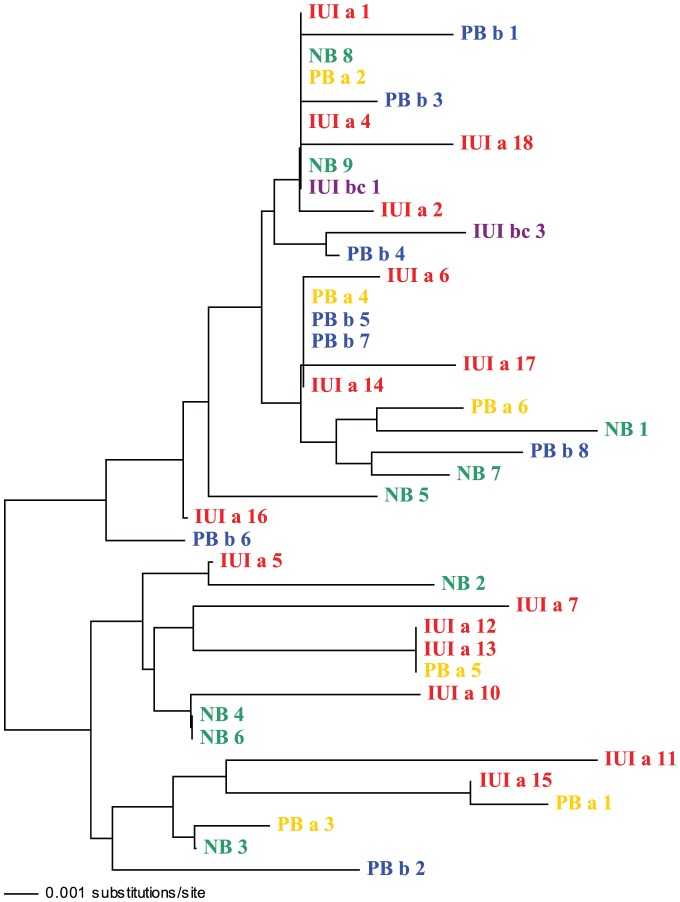
Phylogenetic relationships of recruiting cohorts of *N. myriae*. Clustering is based on a 428 bp sequence of the mitochondrial control region, using the Neighbor-Joining method (Kimura-2 substitution model). Individual recruits belong to one of 5 cohorts: IUIa, IUIb, IUIc, PBa, PBb and NB. Scale bar represents sequence divergence of 0.5%.

At the genetic level, we found no indication of structure among cohorts. Amplification of the hypervariable portion of the control region of 40 *N. miryae* resulted in an alignment of 429 base pairs (bp). There were no indels except for an insertion of a single base pair in a single individual; with all other individuals having regions of 428 bp in length ( GenBank accession numbers: EU447349–EU447388). Out of those 428 bp, 42 were variable, and 21 were phylogenetically informative. Phylogenetic analyses however did not reveal any obvious partitioning of the data (Fig. 2). Consistent with a lack of genetic structure among cohorts, pairwise F_st_ values did not differ significantly from zero (range: 0 to 0.057).

## Discussion

The degree of intra-cohort similarity in otolith micro-chemistry reported here for *N. miryae* provides evidence which suggests that small-scale aggregations of larval fish can appear early in ontogeny and persist through weeks of pelagic dispersal. The dissimilarity among cohorts settling on the same night at nearby sites (∼1 km apart), or one day apart but at the same site, suggests that the high within-cohort similarity is not merely a consequence of local larval retention near shared natal sites. Rather, cohort members appear to move ‘together’ through an environment characterized by small-scale chemical heterogeneity.

Our interpretation of the microchemical data is aided by two related points. First, to the best of our knowledge, there is no documented relationship between the seawater-concentration of the four elements examined in this study (Cr, Cu, Mn & Pb). Second, trace-elemental concentrations in the northern Gulf of Aqaba appear to form a spatially dynamic mosaic of small-scale patches, with significant concentration differences across distances of only several hundreds of meters (Paytan, personal communication).

Together, these two points seem to imply that in order for *N. miryae* cohorts to continuously ‘sample’ similar concentrations of four spatially independent elements, cohort members would have had to be in close proximity throughout the pelagic phase.

Physical forcing cannot be ruled-out as contributing to initial larval cohesion within *N. Miryae* cohorts. However, we suspect that that may not be the case as larval *C. viridis* did not show similar cohesion, despite similarities in: levels of variation in element concentrations (Table 3), breeding habitat, and reproductive mode. Still, other explanations for the lack of pattern in *C. viridis* are possible (e.g. explanations that invoke inter-specific differences in larval bathymetric distribution [23] and larval swimming capabilities [24]), leaving our study mute with respect to the contribution of physical forcing.

The micro-chemical structure among *N. miryae* cohorts was not mirrored at the molecular level. The lack of genetic structure and the selective neutrality of trace-elemental signals argue, in turn, against reproductive sweepstakes or environmental selection as the drivers of cohort composition. As mentioned above, physical forcing is also unlikely to be responsible for non-random cohort composition. Larval behaviour remains the main candidate for explaining the inferred aggregation by of *N. miryae* larvae. Late stage larvae of several benthic fish species are known to school prior to settlement; however, to date, little is known regarding when schooling behaviour begins during the larval phase [11], [25].

Larvae of both *Neopomacentrus* spp. and *Chromis* spp. have been shown, under controlled conditions, to posses the capacity to actively direct their movement – i.e. they are capable, at least towards the end of their development, of orientated [26], fast [27], and prolonged [28] swimming. Difficulties in tracking larvae during the pelagic stage, however, greatly constrain our knowledge of how early, and to what effect, this capacity is translated in situ [29]. Our findings suggest that at least in some species, this capacity is capitalized upon very early in the larval phase; to generate cohesive dispersal.

Clearly, the small sample size of our study (6 *N. miryae* cohorts and 5 *C. viridis* cohorts), as well as its narrow temporal scope, do not capture the full range of temporal and spatial variation expected in the process of marine larval dispersal. Yet, the pattern we describe for *N. miryae* is statistically robust and, even if not representative of a general ‘rule’; it certainly exemplifies the potential for small-scale cohesive dispersal. Moreover, our study exemplifies the insight which is made possible from powerful statistical analysis of otolith-microchemical data (e.g. PaSA [20]), even when lacking direct information on natal sites and their chemical character; and the added value of combining it with the analysis of genetic data.

## Materials and Methods

### Ethics Statement

Samples were taken from juvenile fishes which were collected by the authors a few days after settlement. *N. miryae* were collected from different points along the 7 km long Israeli coast in the northern tip of the Gulf of Aqaba and *C. viridis* were collected from the reef opposite the Interuniversity Institute for Marine Sciences (IUI) at the south part of this coast. All fish were collected using hand nets. Living fishes were not kept for this study. Clove oil was used to anesthetize the fishes which were immediately dropped in 95% Ethanol. One lapilar and one sagittal otolith were removed from each fish. Collections were conducted with permits from the Nature and Parks Authority of Israel. These permits allowed the collection of the specific juvenile fish species, the methods of collection and preservation of the samples and the collection sites (which are under the management the Nature and Parks Authority.

**Figure 3:**
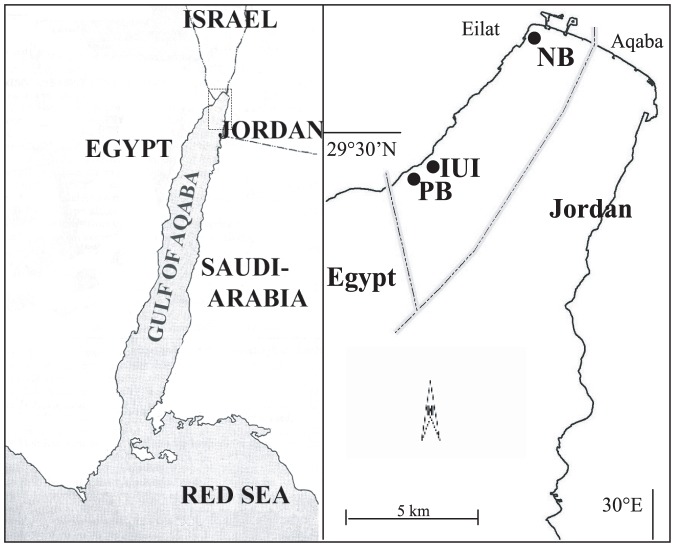
Map of collection sites. *C. Viridis* were collected at IUI. Cohorts IUIa, IUIb and IUIc of *N. myriae* were collected at IUI, cohorts PBa and PBb at PB and cohort NB at NB.

Samples of *N. miryae* were collected using hand nets from their recruitment sites (a natural or artificial vertical structure, with a base of <10 m^2^), a few days after settlement during the winter of 2003/4. The sampled schools were estimated to range between 500 and 5000 fish each. New *C. viridis* recruits were collected from one coral head using clove oil and hand-nets in summer 2005. At the time of collection there were approximately 150 recruits in the sampled coral head. This sample is one of several such samples that we collected for a separate study [18]. All of these samples were similar regarding the variation in settlement dates, and included similarly small cohorts to those analyzed here. For collection sites see Fig. 3.

One lapillar and one sagittal otolith were removed from each of the preserved fish. The former was used to estimate PLD and hatching date based on pre- and post-settlement growth-ring counts, and the latter was prepared for chemical analysis [18], [20]. Trace element concentrations were measured using transverse LA-ICPMS transects through the core [20], [30] and scaled relative to Ca concentration. Profiles spanning 30 ablation-pits were generated for each of 4 elements: Cu, Pb, Mn & Cr. Since otoliths grow from a central core, ablations that progress from the edge of the otolith through the core and on to the opposite edge provide profiles that sample variation in otolith microchemistry twice within each individual. Palindrome Sequence Analysis (PaSA) [20], which examines both halves of the profiles was used to examine the profiles reliability. The method provides a powerful means to separate signal from noise, since only true signals should form a clear palindrome (i.e., show symmetry around the core; [method S1].

Sequence similarity for any two recruits was evaluated using a simple index: *J* = *a*/(*a*+*b*); where *a* and *b* are the number of times an element was ‘present’ in a given ablation pit along the sequence of both or one of the recruits, respectively; summed across ablations and elements. For simplicity, we considered only one side of the palindrome extending from the core to the sulcus (i.e. half of the transect; [methods S1]). For *N. miryae* we considered those sequences generated from the first 11 pits (measured from the core), whereas for *C. viridis* we considered the first 14 pits. Based on microscopic measurements, it is estimated that the distance covered by these sequences coincides roughly with the earlier half of the PLD for *N. miryae* (11 pits≈14 days) and most of the PLD for *C. viridis* [18]. Mean similarities expected for a purely diffusive larval pool were calculated following the randomization of cohort membership across con-specific larvae, and are based on 100 iterations. Mean similarities corresponding to the 5^th^ and 95^th^ percentiles of the expected (null) distribution were used to infer statistical significance (i.e. intra-group similarity greater than the expected by chance).

Shifts in concentration-maxima sequences, which were consistent among the 4 trace elements and are therefore likely to constitute technical artefacts (due to increments' width and aragonite's quality as well as mounting angle), produced ‘empty pits’ that had to be removed to prevent superfluous dissimilarity [methods S1]. To achieve **this, pair-wise similarities of sequences were evaluated using a computerized** algorithm designed to maximize *J* from all the possible shifts of the paired sequences (i.e. all the permutations possible with the removal of 0 to a maximum of 3 empty ablations). In iterations in which the paired sequences did not match in length, *J* was calculated across the length of the shorter sequence. Noteworthy, deviations from perfect similarity (*j* = 1) may be partially attributed to a technical issue associated with the ICPMS used in this study (the Finnigan Element 2 double-focusing sector ICPMS; [methods S1]

The relative concentration of six elements (Mg, Cr, Mn, Sr, Cu & Pb) measured at the core (pit # 0) were evaluated using cluster analysis (separate analysis for each species). Data were normalized prior to analysis, to avoid negative values due to high blank reading (see above). Clustering was based on Euclidean distances and average linkage.

Genetic analysis focused on a sample of 40 *N. miryae*, of which 85% coincided with the individuals used for the micro-chemical analysis (i.e. for technical reasons, not all sampled cohort members were used in the microchemical and/or molecular analyses). Unfortunately, individuals were not stored separately after otolith extraction, so fish could only be assigned to the five cohort groups: IUIa, IUIb&c, PBa, PBb and NB (samples sizes: 15, 2, 6, 8, and 9; respectively). Upon genomic DNA extraction, the hypervariable region of the mitochondrial control region was PCR amplified using the universal primers CR-A L15995 (5′-AATTCTCACCCCTA GCTCCCAAAG-3′) and CR-E H16498 (5′-CCTGAAGTAGGAACCAGATG-3′) [31], and prepared for sequencing, following protocols described by Bay *et al.* [32]. Sequences were verified by comparison against those of other pomacentrids (GENBANK); and analysed using PAUP [33], DNAsp [34], and Arlequin [35]. Fst estimates were estimated using the Kimura 2 parameter model, as implemented in Arlequin (IUIb&c was excluded from the analysis because of small sample size (n = 2)).

## Supporting Information

Methods S1 **Detailed explanation on the way otolith trace-element sequence similarity analysis was conducted.**
(PDF)
