# Supplementary material for: Evidence for Cohesive Dispersal in the Sea
Source: PLoS One. 2012 Sep 18;7(9):e42672. doi: 10.1371/journal.pone.0042672 (PMC3445566; doi:10.1371/journal.pone.0042672)
Supplement: Methods S1 — Detailed explanation on the way otolith trace-element sequence similarity analysis was conducted. (PDF) [file pone.0042672.s001.pdf]

Evidence for cohesive dispersal in the sea

S1 - Supplementary methods - otolith trace-element sequence similarity analysis

The two shoulders (two sides of the core) of the obtained sequence of trace elemental concentrations may differ in the number of pits (ablations) between spikes of high concentrations. For example, the 5 pits of a given element on either side of the core with spikes can be symmetric, like this: 1 0 0 0 1 C 1 0 0 0 1 (C = core); or asymmetric, like this: 1 0 0 0 1 C 1 0 0 1 0. This is due to both natural differences in increment width and to technical difficulties, and thus, obtaining of symmetry in the order of concentrations maxima will be considered as palindrome regardless of asymmetry in the distances of the theses concentrations maxima from the core.

Similarly, differences in the length of the palindrome are expected to be found between otoliths of different fish that have exactly the same palindrome [1]. In addition to the expected differences between the two shoulders of the same otolith, there can be several more causes for potential differences between otoliths: e.g., natural causes, like different qualities of the Aragonite, which can result in differences in the efficiency of the laser; or technical causes, like an otolith mounted with some inclination, resulting in a longer path for the laser through each increment. Even when a good correlation is found between the ablated distance and the number of pits [2], a small change could preclude the possibility to examine similarity based on the raw data (Figure S1).

In order to simplify the comparison, we compared only one shoulder of the otolith (= one side of the palindrome). After the palindrome is identified and the sequence of reliable spikes determined (Figure S2), each side actually contains the entire chemical data. The record of chemical traces starts from the core (which is ideally at the middle of the transect and marked as pit # ‘0’), and continues towards the edges (the more distant, the later). Since there are natural differences in increment width at different sides of the otolith (which may induce additional differences in spike density), we used only those data obtained from the sulcus side, which was the upper side (ablated first) in our samples.

To generate the sequences used for calculating similarity, the concentration profile of each element was coded according to the presence/absence of concentration maxima [1]. Specifically, a non-zero reading that was higher than the one immediately before and after it was scored as ‘1’, while the other two were scored as ‘0’ (Fig. S1)

Differences in the distances (in pits) between spikes obtained in the sequences of different otoliths also remain when only one side of the palindrome is compared. Some of these differences will feature as additional empty pits along the relatively more “spacious” sequence (Figure S1). Such empty pits should be removed in order to eliminate the noise produced by the different transect sizes by frame-shift/s (pit-shift/s) and thus increase the similarity index (*J*). To achieve this, pair-wise similarities of sequences were evaluated using a computerized algorithm designed to maximize *J* from all the possible frame-shifts of the paired sequences (i.e. all the permutations possible

with the removal of 0 to a maximum of 3 empty ablations; Figure S1). In iterations in which the paired, frame-shifted, sequences did not match in length, *J* was calculated across the length of the shorter sequence.

On average, 0.82 (± 0.92) and 1.39 (± 1.10) empty pits were removed from the within and between cohort comparisons, respectively. As the same sequence may be frame-shifted differently when paired with different sequences, we estimated the consistency with which a given empty ablation was removed. Specifically, for each fish we calculated the proportion of pair-wise comparisons from which an empty pit was removed. For those pits that were removed at least once, the proportions ranged from 0.38 to 1.00 (median: 0.69) and 0.07 to 0.76 (median: 0.39); for within and between cohort comparisons, respectively.

Expected mean similarities were generated by the random allocation of fish into the existing cohorts of each species, while preserving the observed cohort size. Percentiles were calculated across 100 iterations of the randomization process.

Fish that have been together and experienced the same environment are expected to have the same sequence [1] and thus *J* values obtained from these fish after being frame-shifted are expected to be 1 or close to it. However, some of the obtained results within a cohort were below this value (although above the expected random values). We attribute some of these findings to an additional technical issue. The machine we used (Finnigan Element 2 double-focusing sector Inductively Coupled Plasma Mass

Spectrometer) automatically deducts the last blank from each count. Thus, when counts are low and the last blank is high, negative counts may ensue and some of the weaker spikes may disappear. Examining the readings below zero revealed that most of these missing spikes in fact exist (Figure S2). Although we have not considered these spikes in our analysis, this factor explains some of the relatively low *J* values found within cohorts.

References

1 Ben-Tzvi, O., Abelson, A., Gaines, S. D., Sheehy, M. S., Paradis, G. L. & Kiflawi, M. 2007 The inclusion of sub-detection limit LA-ICPMS data, in the analysis of otolith microchemistry, by use of a palindrome sequence analysis (PaSA). Limnol Oceanogr-Methods 5, 97-105. (DOI: 10.4319/lom.2007.5.97)

2 Ruttenberg, B. I., Hamilton, S. L., Hickford, M. J. H., Paradis, G. L., Sheehy, M. S., Standish, J. D., Ben-Tzvi, O. & Warner, R. R. 2005 Elevated levels of trace elements in cores of otoliths and their potential for use as natural tags. Mar Ecol Prog Ser **297**, 273-281. (doi:10.3354/meps297273)

Figure legend

A

Fish	El	1	2	3	4	5	6	7	8	9	10	11	12	13	14	15
	Cr	0	1	0	0	0	0	1	0	1	0	1	0	0	0	0
IUI a	Mn	0	0	0	0	0	1	0	0	0	0	1	0	1	0	0
3302	Cu	1	0	0	1	0	0	0	1	0	1	0	1	0	0	0
	Pb	0	1	0	0	0	1	0	1	0	0	0	0	1	0	0
	Cr	0	0	1	0	0	0	0	1	0	1	0	1	0	0	0
IUI a	Mn	0	0	0	0	0	0	1	0	0	0	0	1	0	1	0
3308	Cu	1	0	0	0	1	0	0	0	1	0	1	0	1	0	0
	Pb	0	0	1	0	0	0	1	0	1	0	0	0	0	1	0

B

Fish	El	1	2	3	4	5	6	7	8	9	10	11	12	13	14	15
	Cr	0	1	0	0	0	0	1	0	1	0	1	0	0	0	0
IUI a	Mn	0	0	0	0	0	1	0	0	0	0	1	0	1	0	0
3302	Cu	1	0	0	1	0	0	0	1	0	1	0	1	0	0	0
	Pb	0	1	0	0	0	1	0	1	0	0	0	0	1	0	0
	Cr	0	1	0	0	0	0	1	0	1	0	1	0	0	0	
IUI a	Mn	0	0	0	0	0	1	0	0	0	0	1	0	1	0	
3308	Cu	1	0	0	1	0	0	0	1	0	1	0	1	0	0	
	Pb	0	1	0	0	0	1	0	1	0	0	0	0	1	0	

C

Fish	El	1	2	3	4	5	6	7	8	9	10	11	12	13	14	15
	Cr	0	1	0	0	0	0	1	0	1	0	1	0	0	0	0
IUI a	Mn	0	0	0	0	0	1	0	0	0	0	1	0	1	0	0
3302	Cu	1	0	0	1	0	0	0	1	0	1	0	1	0	0	0
	Pb	0	1	0	0	0	1	0	1	0	0	0	0	1	0	0
	Cr	0	1	0	0	0	0	1	0	0	0	0	1	0	1	0
IUI b	Mn	0	0	0	0	0	0	1	0	1	0	0	0	0	0	0
3324	Cu	0	0	1	0	0	0	1	0	0	0	1	0	0	0	1
	Pb	0	1	0	1	0	0	1	0	0	0	1	0	0	0	0

D

Fish	El	1	2	3	4	5	6	7	8	9	10	11	12	13	14	15
	Cr	0	1	0	0	0	0	1	0	1	0	1	0	0	0	0
IUI a	Mn	0	0	0	0	0	1	0	0	0	0	1	0	1	0	0
3302	Cu	1	0	0	1	0	0	0	1	0	1	0	1	0	0	0
	Pb	0	1	0	0	0	1	0	1	0	0	0	0	1	0	0
	Cr	1	0	0	0	0	1	0	0	0	0	1	0	1	0	
IUI b	Mn	0	0	0	0	0	1	0	1	0	0	0	0	0	0	
3324	Cu	0	1	0	0	0	1	0	0	0	1	0	0	0	1	
	Pb	1	0	1	0	0	1	0	0	0	1	0	0	0	0	

E

Fish	El	1	2	3	4	5	6	7	8	9	10	11	12	13	14	15
	Cr	0	1	0	0	0	0	1	0	1	0	1	0	0	0	0
IUI a	Mn	0	0	0	0	0	1	0	0	0	0	1	0	1	0	0
3302	Cu	1	0	0	1	0	0	0	1	0	1	0	1	0	0	0
	Pb	0	1	0	0	0	1	0	1	0	0	0	0	1	0	0
	Cr	1	0	0	0	1	0	0	0	0	1	0	1	0		
IUI b	Mn	0	0	0	0	1	0	1	0	0	0	0	0	0		
3324	Cu	0	1	0	0	1	0	0	0	1	0	0	0	1		
	Pb	1	0	1	0	1	0	0	0	1	0	0	0	0		

F

Fish	El	1	2	3	4	5	6	7	8	9	10	11	12	13	14	15
	Cr	0	1	0	0	0	0	1	0	1	0	1	0	0	0	0
IUI a	Mn	0	0	0	0	0	1	0	0	0	0	1	0	1	0	0
3302	Cu	1	0	0	1	0	0	0	1	0	1	0	1	0	0	0
	Pb	0	1	0	0	0	1	0	1	0	0	0	0	1	0	0
	Cr	1	0	0	0	1	0	0	0	1	0	1	0			
IUI b	Mn	0	0	0	0	1	0	1	0	0	0	0	0			
3324	Cu	0	1	0	0	1	0	0	1	0	0	0	1			
	Pb	1	0	1	0	1	0	0	1	0	0	0	0			

Figure S1: Sequence similarity analysis. The method and logic of similarity analysis are presented as pairs of sequences. The cohort and serial # of the fish are given in the left column. The highest reading for each element at a spike (spike can occupy more than one pit) is marked ‘1’. All other readings are marked ‘0’. The number of ablations (from the core towards the sulcus) heads each column. Colours are used to track the original pit position after being shifted. Table A: Raw sequence of two fish of the same cohort. Similar spikes are seen in pit 2 of the upper fish and pit 3 of the lower one. There is no similarity between pits along the entire sequence (except for pit # 1) However, deleting pit 2 from the lower sequence will result in a perfect fit, as seen in Table B. Table C: Comparison of two sequences obtained from otoliths of fish from different cohorts. There is no fit between the sequences except for pit # 2 and partial fit in pit # 7. The fit between the sequences is not improved when pit # 1 is deleted from the lower sequence (D) or when pits # 1 & 5 are deleted (E). When a third pit (# 9) is deleted (F) there are two pits which then fit (# 8 & 9 in Table E). To find maximum fit, we allowed removal of pits with no spikes if no similar spike was found on either side. For example pit # 13 in the lower sequence of Table C cannot be deleted since Cr spikes exist on both sides and, thus, by deleting it we would be ignoring one of them and considering only the higher. When two consecutive empty pits exist (e.g. pit # 5 & 6 in the lower sequence of Table C), we allowed the deletion of only one.

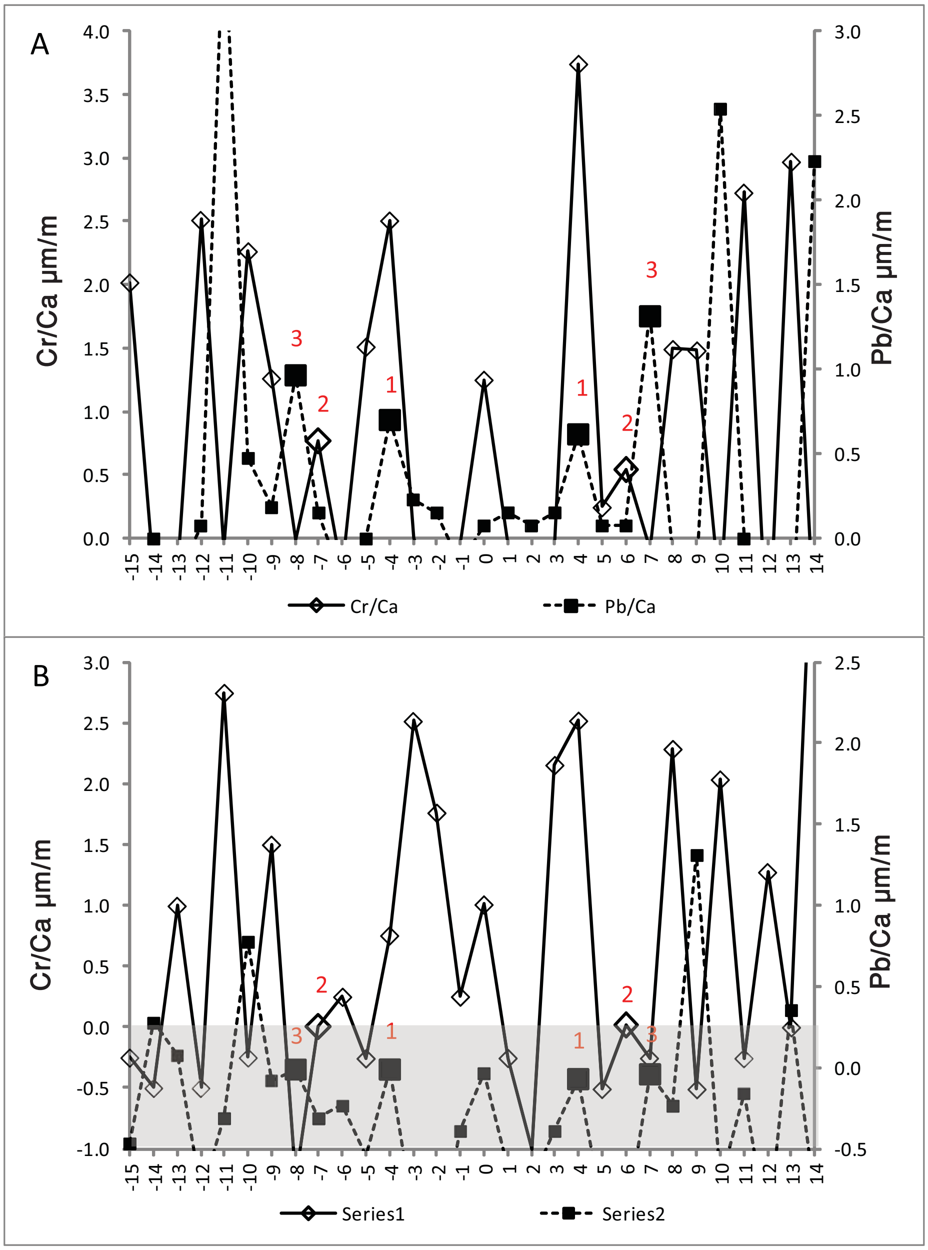

Figure S2: Sample sequence made of two (out of four) elements. Pit # 0 represents the core (which was identified by high concentrations of Mn & Mg2). The distances from the core are given in pits (negative values for pits that were ablated prior to the core). The spikes that are found in the same (mirror) order on both sides of the core form a palindrome. A spike without its pair on the other shoulder is not included in the sequence.

A & B are taken from different N. miryae fish of the same cohort. The sequence is similar except for three spikes (marked with larger symbols and numbered) that were found in the positive zone only in A. The missing spikes, which were found as negative values (the gray area in B; under the last blank which is deleted from each single count by the machine), were not included in the compared sequences and thus explain some of the lower similarities that were obtained from comparisons within the cohort. The method used did not allow the discarding of pits with spikes of more than one element if only one of the elements was missing (e.g. B pits # -3 & 4) and thus kept the dissimilarity obtained due to the negative result of only one element.
